# Burden of Disease in Patients With Tumor‐Induced Osteomalacia

**DOI:** 10.1002/jbm4.10436

**Published:** 2020-12-18

**Authors:** Fernando Jerkovich, Selva Nuñez, Yamile Mocarbel, Analía Pignatta, Natalia Elías, Hamilton Cassinelli, Adriana Graciela Díaz, Carlos Vigovich, María Celeste Balonga, Ana Carolina Cohen, Giselle Mumbach, Sofía Gonzalez, José Rubén Zanchetta, María Belén Zanchetta

**Affiliations:** ^1^ Instituto Diagnóstico e Investigaciones Metabólicas, Universidad del Salvador Buenos Aires Argentina; ^2^ División Endocrinología, Hospital de Clínicas Universidad de Buenos Aires Buenos Aires Argentina; ^3^ Servicio de Endocrinología Hospital Interzonal San Juan Bautista San Fernando del Valle de Catamarca Argentina; ^4^ Servicio de Endocrinología, Metabolismo, Nutrición y Diabetes Hospital Británico de Buenos Aires Buenos Aires Argentina; ^5^ Centro de Investigaciones Endocrinológicas “Dr. César Bergadá” (CEDIE) Consejo Nacional de Investigaciones Científicas y Técnicas (CONICET) – Fundación de Endocrinología Infantil (FEI) – División de Endocrinología Hospital de Niños Ricardo Gutiérrez Buenos Aires Argentina; ^6^ Hospital Gobernador Centeno General Pico Argentina; ^7^ Sanatorio Rivadavia San Miguel de Tucumán Argentina; ^8^ Instituto de Ortopedia y Traumatología Posadas Argentina; ^9^ Hospital Lagomaggiore Mendoza Argentina

**Keywords:** TUMOR‐INDUCED OSTEOMALACIA, HEALTH‐RELATED QUALITY OF LIFE, MUSCLE STRENGTH, BONE PAIN, FATIGUE

## Abstract

Tumor‐induced osteomalacia (TIO) is a chronic condition associated with muscle weakness and long‐term disability. We conducted a cross‐sectional study of patients diagnosed with TIO who had been referred to our institution between May 2018 and December 2019. Our aim was to assess health‐related quality of life (HRQoL), fatigue, pain, and muscle mass and strength in these patients. Detailed information was obtained regarding general characteristics, initial symptoms and biochemical parameters measured at diagnosis and on the first visit to our institution. Fatigue was assessed using the Functional Assessment of Chronic Illness Therapy‐Fatigue (FACIT‐Fatigue) scale, pain using the Brief Pain Inventory–Short Form (BPI‐sf) scale and HRQoL by the 36‐item Short Form survey (SF‐36) questionnaire. Eight patients were included in the study: three without tumor localization, four with nonremission after surgery, and one with clinical recurrence 2 years after surgery. Fatigue experienced by patients with TIO was significantly higher compared to the general population (*p* ˂ .0001). The physical summary measure of the SF‐36 showed significantly lower values than those of the Argentinean population with chronic conditions (mean 20.4 versus 45.9, *p* < .0001). According to the BPI‐sf, patients with TIO have moderate average pain and the pain interferes severely with walking, general activities, work, and mood. Seven patients had a diagnosis of sarcopenia, four of which had severe sarcopenia. To our best knowledge, this is the first study aimed to quantify fatigue, pain, HRQoL, and muscle mass and strength in a group of patients with TIO. We hope our results contribute to a better understanding of the burden of disease and to establish a basis for future studies—with larger samples—which will make it possible to assess the efficacy of therapeutic interventions for these conditions. © 2020 American Society for Bone and Mineral Research © 2020 The Authors. *JBMR Plus* published by Wiley Periodicals LLC on behalf of American Society for Bone and Mineral Research.

## Introduction

Tumor‐induced osteomalacia (TIO) is a rare paraneoplastic syndrome, caused by overproduction of fibroblast growth factor 23 (FGF23)—or other phosphatonins—secreted by a mesenchymal tumor.^(^ ^1^, ^2^ ^)^ The largest cohort from a single center included 230 patients.^(^ ^3^ ^)^ FGF23 excess leads to hypophosphatemia due to renal phosphate wasting and inappropriately normal or low 1.25 dihydroxyvitamin D. As a result, patients often present with a long history of musculoskeletal pain, fatigue, proximal muscle weakness, gait disturbance, and multiple fractures. These debilitating symptoms are a consequence of severe hypophosphatemia and can lead to long‐term disability and considerable morbidity.

Although complete tumor resection results in resolution of symptoms in most patients,^(^ ^3^ ^)^ the diagnosis of TIO is commonly delayed because of its rarity and unspecific symptoms, and because tumor localization remains a challenging task.^(^ ^4^ ^)^

In clinical practice TIO is largely known as a chronic condition that causes muscle quality deterioration and has a significant impact on quality of life (QoL).^(^ ^5^ ^)^ Nevertheless, limited research has been done to quantify the clinical burden of disease in this group of patients. The aim of our study was to evaluate health‐related quality of life (HRQoL), fatigue, pain, and muscle mass and strength in a group of adult patients with TIO referred to our institution.

## Patients and Methods

### Selection of patients

We included adult patients with clinical diagnosis of TIO referred to our institution between May 2018 and December 2019. Diagnosis of TIO was based on the presence of a compatible clinical presentation and hypophosphatemia associated with a low tubular reabsorption of phosphate (TRP), in the absence of a relevant family history. For tumor localization, if it was clinically evident on physical examination, directed images were obtained (CT, MRI, or ultrasonography) to adequately characterize the lesion. Otherwise, functional imaging was performed.

The study was approved by Institutional Ethics Committee: Comité de Ética en Investigación del Instituto Nacional de Psicopatología (CEIINAPsi).

### Clinical characteristics

Detailed information regarding general characteristics (age at diagnosis, gender, height, weight, time from onset to diagnosis, number of physicians seen from onset to diagnosis, time of follow‐up, tumor localization) and initial symptoms (bone and muscle pain, fragility fractures, and mobility impairment) were obtained through a medical interview and clinical records sent by the referring physician.

Clinical status at the time of first visit to our institution was recorded as: (i) patients without tumor localization, (ii) patients with nonremission after tumor resection, (iii) patients with recurrence after tumor resection, and (iv) patients with full recovery after tumor resection.

### Biochemical features and bone mineral density

Biochemical parameters measured at diagnosis and on the first visit to our institution (calcium, phosphate, creatinine, 25‐hydroxyvitamin D, parathyroid hormone, alkaline phosphatase, and TRP) were assessed.

FGF23 levels were measured at our institution by the enzyme‐linked immunosorbent assay using the Human intact FGF‐23 ELISA Kit (MyBioSource Inc., San Diego, CA, USA). We established a normal range of intact FGF23 for healthy persons between 7.5 and 76 pg/mL. According to Endo and colleagues,^(^ ^6^ ^)^ in adults, serum phosphate level lower than 2.5 mg/dL and FGF23 level higher than 30 pg/mL by the intact FGF23 assay indicate the presence of diseases caused by excess actions of FGF23.

Bone mineral density (BMD) (g/cm^2^) was measured by dual‐energy X‐ray absorptiometry (DXA) with GE Lunar Prodigy equipment (GE Lunar, Madison, WI, USA) at the lumbar spine (L_1_–L_4_), femoral neck, and total hip. A *Z*‐score <2 was considered low BMD.

### Fatigue

Fatigue was assessed using the Functional Assessment of Chronic Illness Therapy‐Fatigue (FACIT‐Fatigue) scale.^(^ ^7^ ^)^ The FACIT‐Fatigue scale is a 13‐item questionnaire that assesses self‐reported fatigue and its impact upon daily activities and function. It was initially developed for cancer patients, but it has been validated for use in a variety of chronic illnesses such as rheumatoid arthritis, osteoarthritis, and Gaucher disease.^(^ ^7^ ^)^ A validation in a Spanish‐speaking population was performed by Hahn and colleagues.^(^ ^8^ ^)^ The responses to the 13 items are each measured on a four‐point Likert scale; the total score ranges from 0 to 52, where high scores represent less fatigue. The FACIT‐F scale mean score of the US general population is 43.6 ± 9.4.^(^ ^9^ ^)^ A permission to use this questionnaire was obtained from the authors.

### HRQoL

HRQoL was evaluated using the 36‐item Short Form survey (SF‐36) scale, which was validated in Argentina.^(^ ^10^ ^)^ The SF‐36 is a 36‐item patient‐reported questionnaire that covers eight physical and mental health domains: physical functioning (10 items), bodily pain (two items), role limitations due to physical health problems (four items), role limitations due to personal or emotional problems (four items), emotional well‐being (five items), social functioning (two items), energy/fatigue (four items), and general health perceptions (five items). Scores for each domain range from 0 to 100, with a higher score indicating better HRQoL. To calculate physical (PCS) and mental (MCS) component summary scales, scores for each of the eight domains were standardized using a *Z*‐score transformation, aggregated using factor score coefficients and then multiplied by 10 and added to 50 to generate normalized scores.^(^ ^11^ ^)^

### Pain assessment

Pain was captured using the Brief Pain Inventory Short Form (BPI‐sf),^(^ ^12^ ^)^ which has been validated for use in a Spanish‐speaking population.^(^ ^13^ ^)^ The BPI‐sf is a nine‐item self‐administered questionnaire used to evaluate the severity of a patient's pain and the impact of this pain on the patient's daily functioning. Patients are asked to rate their worst, least, average, and current pain intensity, and rate the degree to which pain interferes with their general activity, mood, walking ability, normal work, relations with other persons, sleep, and enjoyment of life on a 10 point scale. Ratings of 1 to 4 correspond to mild, 5 to 6 to moderate, and 7 to 10 to severe pain intensity and interference with the patient's life.^(^ ^14^ ^)^ BPI‐sf has proved to be a useful tool to assess pain in patients with X‐linked hypophosphatemia (XLH).^(^ ^15^ ^)^

### Muscle mass, muscle strength, and physical performance

Muscle mass was determined in the four limbs (appendicular skeletal muscle mass [ASM]) by DXA. The ASM index was defined as ASM/height^2^ (kg/m^2^). Sarcopenia was defined as <2 standard deviations (SDs) below the sex‐specific mean of young adults (7 kg/m^2^ for men and 5.5 kg/m^2^ for women), according to the latest European consensus.^(^ ^16^ ^)^

Muscle strength was evaluated by hand‐grip strength assessment (Jamar Hydraulic Hand Dynamometer, Patterson Medical, Warrenville, IL, USA) in the dominant hand. The best result of three trials was recorded. According to the European Working Group on Sarcopenia in Older People 2 (EWGSOP2), low strength was defined as ˂16 kg in women and ˂27 kg in men.^(^ ^17^ ^)^

Physical performance was assessed by the 4‐m Walk Gait Speed and Sit to Stand Tests. The 4‐m Walk Gait Speed Test was performed after a practice test and low walking speed was defined as walking slower than 0.8 m/s.^(^ ^17^ ^)^ Participants were instructed to stand with both feet touching the starting line and to begin walking at their usual pace. In the Sit to Stand Test, the time taken for five repetitions without using hands was recorded. Low sit‐to‐stand performance was defined as ˃15 s for five full stands, according to the EWGSOP2.^(^ ^17^ ^)^

### Statistical analysis

Quantitative data are presented as the mean ± SD or the median and range. Categorical data are presented as frequencies and percentages (%). To compare means, the Student's *t* test was used for parametric variables and the Mann‐Whitney *U* test for nonparametric variables. The normal distribution of continuous data was assessed using the Kolmogorov‐Smirnov or the Shapiro‐Wilkes test when appropriate. The Spearman's rank correlation coefficient was used to evaluate the strength of relationship between age at diagnosis, time from onset to diagnosis, phosphate levels, FACIT‐Fatigue score, SF‐36 domains, BPI‐sf values, and muscle health parameters. A *p* value <.05 was considered statistically significant. Statistical analysis was performed using IBM SPSS software, v.20 (IBM Corp., Armonk, NY, USA).

## Results

### Patient characteristics

Eight adult patients with clinical diagnosis of TIO were referred to our institution. Tumor localization was not possible in three. These patients (40, 44, and 48 years old at diagnosis) did not have bone or dental disorders in childhood or a family history compatible with XLH. Tumors with histopathologic confirmation of mesenchymal origin, mixed connective tissue type, were found in feet (*n* = 2), pelvis (*n* = 1), vertebrae (*n* = 1), and head (*n* = 1). Four patients were in nonremission after surgery and one patient had had a recurrence 2 years after surgery.

The clinical and demographic characteristics of the patients in our cohort are detailed in Table 1. Median time from symptoms onset to correct diagnosis was 3.3 years.^(^ ^2^, ^3^, ^4^, ^5^, ^6^, ^7^, ^8^, ^9^, ^10^ ^)^ All of them had been initially misdiagnosed with at least five incorrect diagnosis including osteoporosis, hip osteonecrosis, complex regional pain syndrome, and lumbar pain. A median of seven physicians^(^ ^2^, ^3^, ^4^, ^5^, ^6^, ^7^, ^8^, ^9^, ^10^, ^11^, ^12^, ^13^, ^14^, ^15^ ^)^ had evaluated these patients before reaching the diagnosis of TIO and in all cases the correct diagnosis was finally made by an endocrinologist. All patients presented bone pain as an initial symptom. Muscle pain (87%) and progressive mobility impairment (87%) were also present at onset. Fragility factures had occurred in the majority of patients (87%) and the main sites were: ribs (75%), hip (62%), pelvis (25%), tibia (25%), and vertebrae (12%). All patients had shown low serum phosphorus, reduced TMP and elevated ALP levels at diagnosis and they maintained these features on their first visit to our institution. All patients had high (*n* = 6) or inappropriately normal (*n* = 2) values of FGF23 (median FGF23: 80.8 pg/mL). All of them had low BMD. BMD and *Z*‐score values are described in Table 2.

**Table 1:** Clinical and Biochemical Characteristics of the Eight Patients With Tumor‐Induced Osteomalacia

Characteristic	Value	Reference range
Demographic data		
Gender, female, *n* (%)	5 (62)	
Age at diagnosis (years), median (range)	46 (27–56)	
Age at first visit to our institution (years)	47 (28–65)	
Time from onset to diagnosis (years), median (range)	3.3 (2–10)	
Time of follow‐up (years), median (range)	1.6 (0.8–14)	
Number of physicians seen from onset to diagnosis, median (range)	7 (2–15)	
Height (m), mean ± SD	1.52 ± 0.16	
BMI (kg/m^2^), mean ± SD	27.8 ± 7.8	
Initial symptoms		
Bone pain, *n* (%)	8 (100)	
Fragility fractures, *n* (%)	7 (87)	
Muscle pain, *n* (%)	7 (87)	
Progressive mobility impairment, *n* (%)	7 (87)	
Biochemical features at diagnosis		
Serum calcium (mg/dL), mean ± SD	9.35 ± 0.24	8.5–10.5
Serum phosphate (mg/dL), mean ± SD	1.45 ± 0.22	2.5–4.5
25OHD (ng/mL), median (range)	37.8 (8.4–70.6)	
PTH (pg/mL), median (range)	67 (43–168)	10–65
Creatinine (mg/dL), mean ± SD	1.00 ± 0.69	0.7–1.2
TRP, mean ± SD	0.78 ± 0.07	0.85–1
ALP (IU/L), median (range)	294 (130–1171)	30–120
Biochemical features at first visit to our institution		
Serum calcium (mg/dL), mean ± SD	9.72 ± 0.16	8.5–10.5
Serum phosphate (mg/dL), mean ± SD	2.08 ± 0.6	2.5–4.5
25OHD (ng/mL), median (range)	30.7 (19.9–54.3)	
PTH (pg/mL), median (range)	31.8 (28.4–179.7)	10–65
β‐CTX (ng/mL), median (range)	0.597 (0.135–1.255)	0.07–0.550^a^
TRP, mean ± SD	0.72 ± 0.43	0.85–1
Bone specific ALP (U/L), median (range)	61.7 (63.8–92.6)	˂21.3
iFGF23 (pg/mL), median (range)	82.5 (69–150)	47.5–76

25OHD = 25‐hydroxyvitamin D; ALP = alkaline phosphatase; iFGF23 = intact fibroblast growth factor 23; PTH = parathyroid hormone; SD = standard deviation; TRP = tubular reabsorption of phosphate; β‐CTx = C‐terminal cross‐linked telopeptide of type I collagen.

^a^ Normal reference for premenopausal women: 0.074–0.550; for 30‐year‐old to 50‐year‐old men: 300 ± 142.

**Table 2:** BMD of Patients in Our Cohort

BMD	Value
Spine L_1_–L_4_ BMD (g/cm^2^), mean ± SD	0.856 ± 0.19
Spine L_1_–L_4_ *Z*‐score, mean ± SD	−2.6 ± 1.3
Left total hip BMD (g/cm^2^), mean ± SD	0.712 ± 0.09
Left total hip *Z*‐score, mean ± SD	−2.8 ± 1.6

BMD = bone mineral density; SD = standard deviation.

### Fatigue, HRQoL, pain, and muscle assessment

The mean FACIT‐Fatigue score in our cohort was 28.4 ± 9.6 (25 ± 3.5 in men and 31 ± 12.6 in women) (Table 3). These values were significantly lower in comparison with those of the US general population (*p* ˂ .0001), which means patients with TIO show higher levels of fatigue. The questions that reflected higher level of fatigue (50% of the cohort answered “very much” or “quite a bit”) were: “I have to limit my social activity because I am tired” and “I have trouble starting things because I am tired.”

**Table 3:** Fatigue, Pain, and Health‐Related Quality of Life Assessment

Assessment	Our cohort (*n* = 8)	General population^a^	*p*
FACIT‐F	28.4 ± 9.6	43.6 ± 9.4	˂.0001^b^
SF‐36			
Physical functioning	18 ± 25	89.1 ± 14.4	˂.0001^b^
Physical role functioning	0	83.7 ± 30.4	˂.0001^b^
Bodily pain	27.8 ± 6	80.6 ± 22	˂.0001^b^
General health	44 ± 9.6	71.5 ± 16.5	˂.0001^b^
Energy/fatigue	47 ± 16	62.6 ± 19.8	.0183^b^
Emotional well‐being	61.6 ± 8.3	67.3 ± 19	.3964
Role limitations due to emotional problems	60 ± 43.5	78.9 ± 33.2	.1083
Social functioning	40.2 ± 16.5	81.14 ± 22.5	˂.0001^b^
Physical summary measurements	20.4 ± 8	45.9 ± 11.4	˂.0001^b^
Mental summary measurements	46.4 ± 6	49.9 ± 10	.352
BPI‐pain severity score			
Average	5 ± 2.4		
Worst	6 ± 2		
Least	3.6 ± 1.7		
Pain now	3.8 ± 2.1		
BPI‐interference score			
General activity	8.5 ± 1.6		
Walking	9 ± 1		
Work	8.6 ± 4.2		
Sleep	5 ± 3.8		
Relations with others	4.7 ± 4.3		
Enjoyment of life	6.7 ± 3.2		
Mood	7 ± 2.4		

Values are expressed in means ± SD.

BPI = Brief Pain Inventory; FACIT‐F = Functional Assessment of Chronic Illness Therapy‐Fatigue; SD = standard deviation; SF‐36 = 36‐item Short Form survey.

^a^ For FACIT‐F scores, US general population was used,^(^ ^7^ ^)^ and for SF‐36, Argentinean general population was considered.^(^ ^8^ ^)^

^b^ Statistically significant values.

The physical SF‐36 summary measure showed significant lower values in comparison with an Argentinean population with chronic conditions (mean: 20.4 versus 45.9, *p* < .0001) but the mental summary measure was not statistically different from this reference population (mean: 46.4 versus 49.9, *p* = 0.352). All physical domains of the SF‐36 were statistically lower than in the general population. The domains with the lowest scores were: physical role functioning (which evaluates limitations in usual role activities due to physical health problems), followed by physical functioning and bodily pain (Table 3). Although the mental summary measure was not statistically different from the reference population, specific mental domains such as energy/fatigue and social functioning had lower scores than the general population (47 versus 62.6, *p* = .0183 and 40.2 versus 81.14, *p* ˂ .0001, respectively). No differences with the general population were found regarding the domains of emotional well‐being and role limitations due to emotional problems (Table 3).

According to the BPI‐sf, patients had moderate average pain (mean 5 ± 2.4) and the pain interfered severely with walking (mean 9 ± 1), general activities (mean 8.5 ± 1.6), work (8.6 ± 4.2), and mood (7 ± 2.4) (Table 3). The most frequent sites of pain were hips (50%), knees (37%), and back (25%). The pain was mainly described as tiring, exhausting, sharp, and deep.

Table 4 summarizes the muscle assessment of the eight patients with TIO. Overall, seven patients had a diagnosis of sarcopenia (low muscle strength plus low muscle mass), four of whom with severe sarcopenia (low muscle strength, low muscle mass, and low physical performance). Three patients were unable to stand up from a chair without assistance. In the remaining patients, at least one physical performance test was negatively affected.

**Table 4:** Muscle Mass, Strength, and Physical Performance Assessment

	Women (*n* = 5)	Men (*n* = 3)	Total (*n* = 8)
Muscle mass			
ASM index (kg/m^2^), mean ± SD	5 ± 1.9	7.3 ± 0.1	—
Low muscle mass, *n* (%)	3 (60)	1 (34)	4 (50)
Muscle strength			
Hand grip (kg), mean ± SD	11 ± 10.3	31 ± 1.4	—
Low muscle strength, *n* (%)	4 (80)	1 (34)	5 (62)
Physical performance			
Low walking speed test, *n* (%)	5 (100)	2 (67)	7 (87)
Low sit‐to‐stand test, (%)	4 (80)	3 (100)	7 (87)

ASM = appendicular skeletal muscle mass; SD = standard deviation.

Lower FACIT‐Fatigue values significantly correlated with lower scores in the physical functioning domain of the SF‐36 (ρ = 0.836, *p* = .038) and with higher interference of pain with general activity (ρ = −0.713, *p* = .047). No other significant correlation was found between age at diagnosis, time from onset to diagnosis, phosphate levels, FACIT‐Fatigue score, SF‐36 domains, BPI‐sf values, and muscle parameters.

## Discussion

Our study describes the extremely high clinical burden of disease on eight adult patients with TIO referred to our institution. This cohort showed higher levels of fatigue than a reference population from the United States, lower values in the physical domains of the SF‐36 than in an Argentinean population with chronic conditions and a severe interference of pain with walking, general activities, work, and mood. Regarding muscle assessment, in nearly all patients, at least one physical performance test was negatively affected.

A possible explanation for this clinical picture could be the delay in diagnosis, which in our cohort had taken up to 10 years (median value 3.3 years). Additionally, all our patients had been misdiagnosed and a median of seven physicians had evaluated them before reaching a definitive diagnosis of TIO. This is consistent with the literature, where the reported time from onset of symptoms to a presumptive diagnosis often exceeds 2.5 years and the mean time span from the presumptive diagnosis to tumor localization is 5 years.^(^ ^4^ ^)^

Moreover, none of our patients were cured at the time of their first visit to our institution. This is contrary to what was reported by González and colleagues,^(^ ^18^ ^)^ another case series of patients with TIO in Latin America, where all patients (*n* = 6) were cured after tumor excision in a 6‐month follow‐up period. Similarly, other case reports and case series of patients in Latin America describe successful excision of the tumor responsible for TIO.^(^ ^19^, ^20^ ^)^ It should be noted that our research did not focus on tumor localization and patients were referred to our institution for research purposes from different healthcare levels, revealing the real‐life scenario of patients with TIO across the country.

Fatigue is one of the main symptoms in patients with TIO and it is related to muscle weakness due to longstanding hypophosphatemia.^(^ ^4^ ^)^ Its assessment is complex because psychological and physiological factors may be involved. The FACIT‐Fatigue scale is a validated tool that assesses self‐reported fatigue, with higher scores representing less fatigue. In our cohort of patients with TIO, the FACIT‐Fatigue mean score was 28.4 ± 9.6, which was significantly diminished in comparison to that of the US general population (mean 43.6 ± 9.4, *p* ˂ .0001).^(^ ^9^ ^)^ Unfortunately, the FACIT‐Fatigue scale has not been validated in our country. It is worth mentioning that Mallinson and colleagues^(^ ^21^ ^)^ reported that the FACIT‐Fatigue scores in the range of 30 and below were related to an increased difficulty in performing everyday activities such as folding laundry or getting dressed. In line with these results, the subitem “fatigue/energy” in the SF‐36 score in our patients was significantly affected in comparison with the Argentinean population with chronic conditions (47 versus 62.6, *p* = .0183) and the limitations in usual role activities because of physical health problems showed the worst measurement in this scale. Moreover, the FACIT‐Fatigue values correlate positively with physical functioning domain in the SF‐36 scale (ρ = 0.836, *p* = .038). The FACIT‐Fatigue values in our cohort were similar to the mean score reported in cancer patients with the lowest performance status in the Eastern Cooperative Oncology Group Performance Status (grade 3 and 4, mean value 23.1).^(^ ^9^ ^)^ Grades 3 and 4 of this scale represent patients capable of only limited self‐care, confined to bed or chair more than 50% of waking hours or completely disabled, respectively.^(^ ^22^ ^)^

Our results clearly show that patients with TIO had poor HRQoL in comparison with the general population. Differences were particularly notable in the physical domains of the SF‐36. Besides, the energy/fatigue and social functioning domains were also lower than in the general population. An impaired HRQoL was also found in patients with XLH (a genetic form of a FGF23‐related hypophosphatemic disease).^(^ ^23^, ^24^, ^25^ ^)^ XLH patients showed mean values of SF‐36 score similar to patients with chronic musculoskeletal diseases such as rheumatoid arthritis or juvenile idiopathic arthritis.^(^ ^25^ ^)^ Indeed, in our population, the physical summary measurements were significantly lower even in comparison with a population with chronic conditions in Argentina. Some mental domains (but not the mental summary measurements) were also negatively affected in our cohort. A similar pattern of HRQoL was found in patients with rheumatoid arthritis and osteogenesis imperfecta,^(^ ^26^, ^27^ ^)^ denoting the multifaceted impact of HRQoL on patients with chronic conditions.

Bone pain was reported in nearly 100% of patients with TIO.^(^ ^3^, ^4^ ^)^ In fact, all patients in our study presented with bone pain as an initial symptom. Consistent with the FACIT‐Fatigue and the SF‐36 scores, pain in patients with TIO had higher interference with physical activities including working and walking. Besides, a severe interference with mood and a moderate interference with enjoyment of life were also detected, which might suggest that the psychological and emotional impact of pain needs to be addressed when treating patients with TIO.

It has been shown that hypophosphatemia resulting from vitamin D deficiency is the cause of muscle weakness in patients with osteomalacia.^(^ ^28^ ^)^ It is not definitely clear why phosphate deficiency causes muscle weakness but it is most likely due to the metabolism of ATP, phosphorylation of myosin and actin filaments, alteration in ion pumps and calcium handling, or changes in mitochondrial function.^(^ ^29^ ^)^Although chronic hypophosphatemia is a well‐known factor of skeletal muscle weakness, muscle strength and physical performance have not been quantified in patients with hypophosphatemic disorders. In our study, nearly all patients showed low physical performance denoting a severe compromise in muscle quality.

This study has several limitations. First, the questionnaires we used are not targeted for this specific condition and the small sample size did not allow us to make extensive internal and external validations of the instruments used. Indeed, we could not find any significant correlation between clinical factors and the different scales used in our study, probably due to the low number of patients included. Second, although the study was not limited to patients without tumor localization or without remission, we did not have the chance to evaluate patients with clinical remission after surgery which might have given us a wider picture of the disease. We also did not have a control group to compare their measurements with those of our cohort. Third, the patients in our cohort were evaluated at a reference center and thus, they might have been more severely affected, showing a higher rate of complications than the overall population (referral bias).

To our best knowledge, this is the first study aimed to quantify fatigue, pain, HRQoL, and muscle health in a group of adult patients with non‐cured TIO. According to our results, it seems that this disease has a severe negative impact on patients' QoL, similar to that of advanced cancer. We hope our results contribute to a better understanding of the burden of disease on TIO patients and establish the basis for future studies—with larger samples—which will make it possible to assess the efficacy of therapeutic interventions.

## Disclosures

MBZ has received honoraria from Ultragenyx for participation in an advisory board. HC is a member of the XLH DMP Steering Committee and received honoraria from Ultragenyx for participation in an advisory board. The rest of the authors declare no potential conflicts of interest of this work.

## Author Contributions

**Fernando Jerkovich:** Formal analysis; writing‐original draft. **Selva Nuñez:** Data curation. **Yamile Mocarbel:** Data curation. **Analía Pignatta:** Data curation. **Natalia Elías:** Data curation. **Hamilton Cassinelli:** Data curation. **Adriana Diaz:** Data curation. **Carlos Vigovich:** Data curation. **María Balonga:** Data curation. **Ana Cohen:** Data curation. **Giselle Mumbach:** Data curation. **Sofía Gonzalez:** Data curation. **Jose Zanchetta:** Data curation. **Maria Zanchetta:** Conceptualization; data curation; formal analysis; supervision; writing‐review and editing.

## Authors' roles

MBZ collected and analyzed the data, designed the study and revised the manuscript. FJ analyzed and interpreted the data and drafted the manuscript. SN, YM, AP, NE, HC, AD, CAV, CB, CC, GM, SG, and JRZ collected the data. MBZ and FJ are responsible for the integrity of the data analysis. All authors read and approved the final manuscript.

### Peer Review

The peer review history for this article is available at https://publons.com/publon/10.1002/jbm4.10436.
